# Current Therapeutic Approach to Hypertrophic Scars

**DOI:** 10.3389/fmed.2017.00083

**Published:** 2017-06-20

**Authors:** Zrinka Bukvić Mokos, Anamaria Jović, Lovorka Grgurević, Ivo Dumić-Čule, Krešimir Kostović, Romana Čeović, Branka Marinović

**Affiliations:** ^1^Department of Dermatology and Venereology, University Hospital Centre Zagreb, School of Medicine, University of Zagreb, Zagreb, Croatia; ^2^Laboratory for Mineralized Tissues, School of Medicine, University of Zagreb, Zagreb, Croatia

**Keywords:** wound healing, skin scarring, hypertrophic scar, scar management, topical therapy, prevention, treatment

## Abstract

Abnormal scarring and its accompanying esthetic, functional, and psychological sequelae still pose significant challe nges. To date, there is no satisfactory prevention or treatment option for hypertrophic scars (HSs), which is mostly due to not completely comprehending the mechanisms underlying their formation. That is why the apprehension of regular and controlled physiological processes of scar formation is of utmost importance when facing hypertrophic scarring, its pathophysiology, prevention, and therapeutic approach. When treating HSs and choosing the best treatment and prevention modality, physicians can choose from a plethora of therapeutic options and many commercially available products, among which currently there is no efficient option that can successfully overcome impaired skin healing. This article reviews current therapeutic approach and emerging therapeutic strategies for the management of HSs, which should be individualized, based on an evaluation of the scar itself, patients’ expectations, and practical, evidence-based guidelines. Clinicians are encouraged to combine various prevention and treatment modalities where combination therapy that includes steroid injections, 5-fluorouracil, and pulsed-dye laser seems to be the most effective. On the other hand, the current therapeutic options are usually empirical and their results are unreliable and unpredictable. Therefore, there is an unmet need for an effective, targeted therapy and prevention, which would be based on an action or a modulation of a particular factor with clarified mechanism of action that has a beneficial effect on wound healing. As the extracellular matrix has a crucial role in cellular and extracellular events that lead to pathological scarring, targeting its components mostly by regulating bone morphogenetic proteins may throw up new therapeutic approach for reduction or prevention of HSs with functionally and cosmetically acceptable outcome.

## Introduction

Skin is the largest organ in the human body that is in constant contact with the environment with its primary role to adapt to stresses and tension and to protect other systems within the body. When injured, it must rapidly repair itself to maintain the cutaneous integrity and its external defense function. As a response to injury, at the site of tissue disruption, the highly organized process of wound healing instantly begins and ultimately results in the formation of a scar that never obtains the flexibility or the strength of the original tissue (1). The fact that in the developed countries about 100 million people per year form a dermal scar as a consequence of elective operations or injuries puts this problem among the most common in modern medicine and represents a huge cost to each health system (2, 3). We can expect around 30% of these to undergo abnormal growth due to aberrations in physiologic healing that result in hypertrophic scar (HS) or keloid formation, which are frequently accompanied by a number of esthetic, functional, and social impairments and may lead to decreased quality of life (4). Normotrophic, atrophic, hypertrophic, and keloid scars are all various types of scars with its different clinical appearance, etiology, and pathogenesis, demanding different therapeutic approach. The apprehension of regular and controlled physiological processes of scar formation is of the utmost importance when facing hypertrophic scarring, its pathophysiology, prevention, and therapeutic approach.

## Methodology

In preparing this work, we used PubMed, Google Scholar, and Web of Science to perform literature searches on HS-related research. Key terms used in the search were “scarring,” “wound healing,” “hypertrophic scar,” “scar management,” “scar prevention,” and “scar treatment.” Review articles were used as an initial source of information and, where relevant, information from primary research papers was obtained.

## Wound Healing and Scar Formation

When it comes to deep skin damage, the wound heals in a highly regulated series of dynamic and physiological processes involving various cells, matrix molecules, cytokines, and mediators (5). Wound healing is divided into continuous and overlapping phases including coagulation, inflammatory response phase (the first 48–72 h after the injury); proliferation phase that includes the formation of extracellular matrix (ECM), angiogenesis, and re-epithelization (days 4–21); and final remodeling or maturation phase, which may last up to a year (6, 7). This final regeneration phase results in the formation of a scar with excess collagen and an absence of cutaneous fat and hair follicles (1). Fibrillar collagen, as a main structural component of the ECM, has a crucial role both for the elasticity and the strength of an intact skin and scar tissue (8). Both normal and pathological scars are the result of deposition of collagen type I and III, although collagen synthesis in HSs is two to three times as much as in normotrophic scars (9). Collagen III increases more than type I in the early stages of wound healing but decreases during maturation phase to normal levels (10).

## The Critical Role of Myofibroblasts and Other ECM Components

It is suggested today that it is the ECM that has the critical role in the scar formation (7). Major players involved in the ECM production are fibroblasts, myofibroblasts, transforming growth factor-beta (TGF-β), proteoglycans—decorin, laminin, and fibronectin, matrix metalloproteinases (MMPs), and bone morphogenetic proteins (BMPs) (7). A key role in the formation of dense collagen matrix during the maturation phase belongs to myofibroblasts originating from fibroblasts, which disappear by apoptosis during normal wound healing when epithelialization occurs (11–13). Various biological properties of both fibroblasts and myofibroblasts have profound impact on the progression and regression of HS. These complex processes are influenced by a signaling network involving different cytokines and growth factors of which are to mention, TGF-β, epidermal growth factor, platelet-derived growth factor, connective tissue growth factor (CTGF), and vascular endothelial growth factor, which is known as a key factor in angiogenesis essential for wound healing (14, 15). The transition from fibroblasts to myofibroblasts expressing α-smooth muscle actin (SMA) is influenced by cytokines, previously listed growth factors, especially TGF-β whose activity diminishes upon the completion of wound repair, mechanical stress, and cellular fibronectin (16). Myofibroblasts are responsible for the production of type I and III collagen, secretion of profibrotic cytokines, remodeling of an immature ECM, and wound contraction (12, 16, 17). Additionally, they produce MMPs that catalyze the hydrolysis of the main components of ECM as well as the activity of cytokines and growth factors (18, 19). Degradation of fibrillar collagen type I, II, and III is mediated by specific collagenases-1, 2, and 3 (MMP-1, 8, and 13) and gelatinases MMP-2 and MMP-9 (18). MMPs transcription is not only induced by glucocorticosteroids and interleukin (IL)-1 but also regulated by TGF-β and insulin-like growth factor-1 (20, 21). MMPs expression is low in intact skin, but after injuring their expression is increased (19). It has been demonstrated that inhibitors of MMPs slow wound healing *in vivo*, which indicates that the MMPs are the key regulators of many wound healing processes (19, 22). One of the most important MMPs for the formation of fibrous tissue is procollagen C proteinase-1, BMP1, of the BMP family, which cleaves the carboxylic pro-domain of procollagen I, II, and III to form the insoluble fibrillar collagen of exceptional tensile strength (23–25). Although isolated with other BMP molecules due to their affinity for the heparin, BMP1 does not share the same amino acid sequence homology with other BMPs so it is not an authentic member of the TGF-β superfamily. It belongs to the astacin/BMP1/tolloid-like family of zinc MMPs that are fundamental in the development and formation of the ECM (23, 26). The importance of BMP1 protein was stressed two decades ago by finding that *Bmp1*^−/−^ mice die shortly after birth from the failure of ventral body wall closure due to abnormal collagen fibrillogenesis (27). To overcome issues of early lethality and functional redundancy in *Bmp1*^−/−^ mice, Muir et al. (28) recently utilized *BT^KO^* mice with floxed *Bmp1* and *Tll1* alleles and they came to the findings that loss of the BMP1 proteinase activity resulted in delayed wound healing and significantly thinned and fragile skin with unusually densely grouped collagen fibrils. Their experiment confirmed BMP1-like proteinases as essential proteins to the structure and wound healing of the skin. BMP1 proteinases are crucial for the formation of ECM not only by direct influence to its formation but also indirectly by activating TGF-β superfamily members including BMP-2 and BMP-4, profibrotic TGF-β1, and growth and differentiation factors GDF-8/-11 and IGF (24, 26). To date, there are seven different isoforms of the BMP1 protein (25). A substantial progress in the field of fibrotic diseases in human has been made by findings of a number of BMP1 isoforms at the protein level in the circulation of patients with a variety of fibrotic conditions such as chronic kidney disease, acute bone fracture, acute myocardial infarction, but most importantly BMP1-3 isoform known as mammalian tolloid that circulates as an active enzyme in plasma samples of healthy individuals in lower concentrations (29, 30). Grgurevic et al. (29) utilized their findings of BMP1-3 protein and tested its effect in rats with chronic kidney disease where administration of rhBMP1-3 increased fibrosis, while BMP1-3 neutralizing antibody reduced it and was associated with low plasma levels of TGF-β1, CTGF, and decreased expression of decorin, suggesting that this pathway may be therapeutic target for fibrosis. Decorin is a small leucine-rich proteoglycan produced by myofibroblasts that regulates collagen fibrillogenesis, inhibits the proliferation of fibroblasts, and reduces production of TGF-β1 and collagen synthesis in HS fibroblasts whose production is here significantly increased (31–33). Another important proteoglycan that is produced by myofibroblasts, as well as by keratinocytes, endothelial cells, and dermal fibroblasts is cellular fibronectin. It is responsible for the formation of stable collagen I/III fibrillar network through a mechanism involving integrins (34) but it is also vital for regulating the neovascularization of granulation tissue (35).

Most recently, a breakthrough study that identifies another consequential role of myofibroblast was published (36). Although it has been thought that they are differentiated, this study showed that adipocytes may be regenerated from myofibroblasts during wound healing through activation of adipocyte transcription factors expressed during development, triggered by crucial BMP signaling from the actively growing hair follicles. These findings fortify the importance of BMPs during wound healing and scar formation and identify the myofibroblasts as a plastic cell type that may be manipulated to treat scars in humans.

## Molecular Biology of Wound Healing

Transforming growth factor-beta/Smad signaling has a pivotal role in scar-mediated healing. Both TGF-β1 and TGF-β2 enhance scarring, i.e., promote fibrosis, whereas TGF-β3 reduces scarring; they act through binding to dimeric TGF-β receptor complexes (5, 37, 38). Upon activation, this receptor complex phosphorylates Smad2 and Smad3 proteins, which subsequently form dimers with Smad4 that are able to translocate to nucleus and act as a transcription factor that triggers target gene transcription including collagens I and III (39). TGF-β1 and TGF-β2 activate this dimeric receptor complex and thereby downstream Smad signaling, whereas TGF-β3 is a receptor antagonist that inhibits signal transduction (20, 40). Another Smad protein, Smad7, is thought to prevent Smad2/3-receptor interaction and subsequent phosphorylation that makes Smad7 as the negative feedback regulator of this profibrotic signal pathway. Inducing Smad 7 may be promising way to inhibit fibrosis and prevent HS formation (41). Not only that TGF-β influences collagen production directly through Smad signaling but also induces Smad 3 to transcribe proteins that activate the Wnt pathway that induces scarring (37, 42). It has been experimentally shown that targeting TGF-β/Smad pathway influences fibroblast proliferation and ECM deposition in HS (40, 43–45).

The local healing process is also influenced by systemic response to injury whereby increases in Th2 and possibly Th3 response cytokines such as IL-2, IL-4, IL-10, and TGF-β are found in the circulating lymphocytes in fibrotic conditions (46). Among the other momentous mediators of scarring, there are proinflammatory cytokines IL-6 and IL-8 that enhance scarring, and anti-inflammatory cytokine IL-10 that has the opposite effect (47).

We can say that the key to controlled scarring is a balance between proliferative processes in proliferative phase and degradation and remodeling processes in the early stage of maturation. Thus, the imbalance between proinflammatory, profibrotic growth factors such as TGF-β1 and 2 on one side, and antifibrotic factors such as TGF-β3 and MMPs on the other side, results in overabundant wound ECM or the formation of a HS. Under certain conditions, primarily due to imbalance of synthesis and degradation of collagen, normal scar is replaced by pathological fibrous tissue with decreased or absent cutaneous fat and hair follicles, containing the same ECM molecules as the tissue they replace, but in different ratios; increased production of collagen type I and III, fibronectin, and laminin, and decreased expression of the hyaluronic acid and decorin (7, 47, 48).

## HSs Versus Keloids

**Figure 1:**
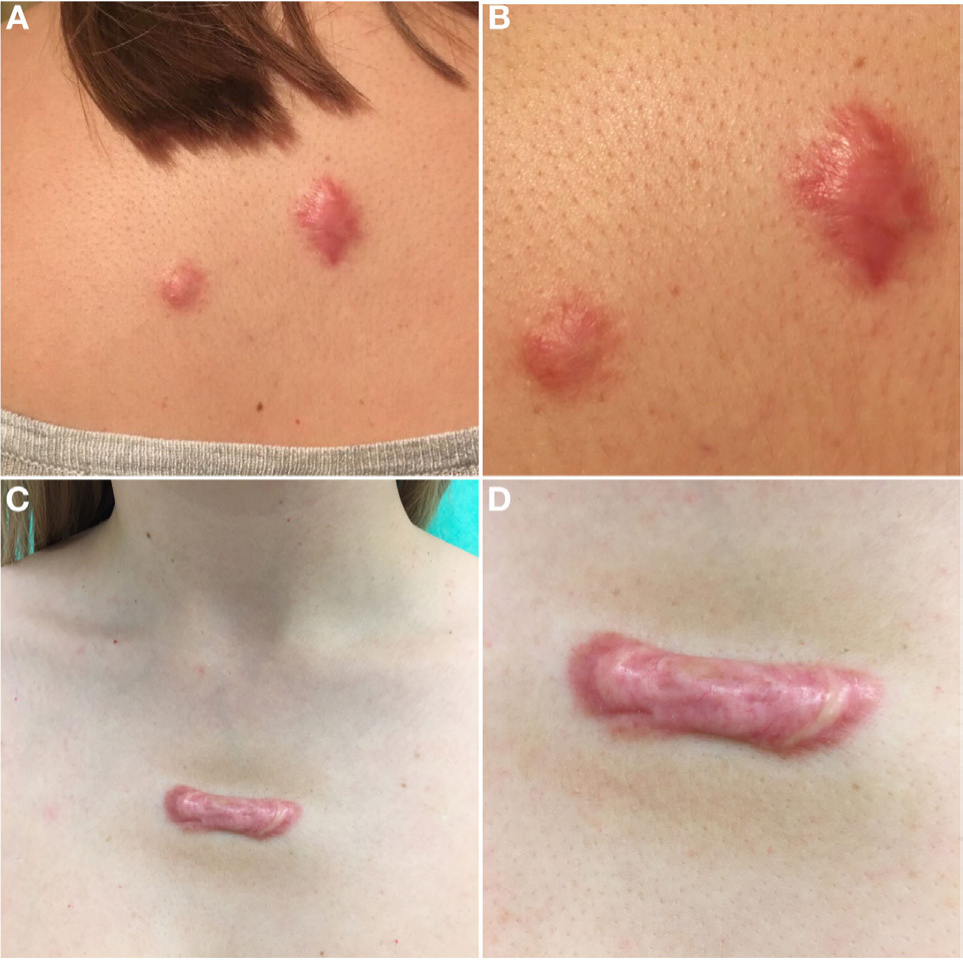
Clinical appearance of hypertrophic scar (HS) and keloid. **(A)** HSs on upper back that developed after excision of dermal nevi. **(B)** Close-up of HSs. **(C)** Chest of patient showing keloid developed spontaneously. **(D)** Close-up of keloid.

Hypertrophic scars mostly develop within 1–3 months after deep skin injury, surgical procedure or burns, in contrast with keloids that may occur up to 12 months after injury or even develop spontaneously (49). Many factors such as age, genetic factors, race, hormone levels, and immunologic responses of the individuals appear to play a role (50–52). Not least important, are the type of injury, wound size and depth, anatomic region, and mechanical tension on the wound (20). Ogawa and Akaishi (51) proposed that all of this mentioned risk factors promote pathological scar formation by inducing endothelial dysfunction (i.e., vascular hyperpermeability) that prolongs and intensifies inflammation, thereby leading to fibroblast dysfunction. The first challenge when dealing with pathological scarring in daily clinical practice is a classification of a scar. Scars can be classified as mature, immature, linear hypertrophic, widespread hypertrophic, minor and major keloid (53). The diagnosis is usually clinical based upon scar appearance, etiology, and growth pattern. Based on current guidelines (54), immature scars are morphologically red and raised, are often associated with slight pain and pruritus, and evolve into mature scars that are pale, soft, narrow, and flat. Linear HSs are those scars that we use as a model of HS type with all of their typical characteristics described in Table 1. Extensive HSs present with irregular, highly erythematous surface and have hardened cord-like appearance. They are usually caused by thermal or chemical burns and lead to functional impairment due to contractures. The terms HS and keloid are often used inconsistently and interchangeably. Although there are clinical similarities between two of them, there are many pathological and biochemical differences that suggest that these entities are distinctive (55, 56). HSs are characterized as raised, pink or red scars, sometimes pruritic and painful, within the margins of the original wound (Figures 1A,B), that develop soon after surgery and usually subside with time as opposed to keloids (Figures 1C,D) that spread out of the margins of the wound, may develop months after the trauma, and continue to evolve over time without regression (49, 57). HSs and keloids are also distinguishable based on their histologic characteristics. HSs contain primarily type III collagen bundles that are oriented parallel to the epidermal surface arranged in a wavy pattern with abundant nodules containing myofibroblasts expressing α-SMA and large extracellular collagen filaments. In contrast, keloid tissue is composed of disorganized type I and III thick, eosinophilic collagen bundles that appear randomly oriented to the epithelial surface with no nodules or excess myofibroblasts (9, 56, 58, 59). To note is that HSs go through a remodeling phase, while keloids do not enter this final wound healing phase. Remodeling happens due to the presence of myofibroblasts in HSs that account for various processes during this phase and contraction of the wound. HS myofibroblasts are less responsive to apoptotic signals and produce more ECM components especially type I collagen, whose synthesis is seven times higher than normal (56). Treatment of HS is demanding, often painful, enduring, and mostly unsatisfactory (3). Due to the similar underlying pathophysiology, HSs and keloids may respond to the same treatment modalities. However, HSs are often more responsive and less prone to recurrence, which makes them therapeutically less challenging. To date, multiple invasive and non-invasive therapies have been used and proposed, but none of these has been adequately evaluated in high-quality studies (60). Management of HS has transitioned from invasive methods to intralesional and topical therapies that act at a cellular level (61).

**Table 1:** Differences in HSs and keloids.

HSs	Keloids
Frequent incidence	Rare incidence
Posttraumatic	Posttraumatic or spontaneous
Develop soon after surgery	May not begin for many months
Usually subside with time	Rarely subside with time
Remain within the wound boundaries	Spread outside the wound boundaries
No predominant anatomical site but often occur when skin creases are at right angle or when scars cross joints	Predominant anatomical sites (chest, shoulders, upper back, earlobes, posterior neck, knees)
Pruritic, rarely painful	Pruritic, painful
Less association with phototype	More common in darker skin types
Genetic predisposition	Less genetic predisposition
Improve with appropriate surgery, low recurrence rate	Often worsened by surgery, high recurrence rate
Increase collagen synthesis; 7 times higher than normal	Increase collagen synthesis; 20 times higher than normal
Collagen type I < III	Collagen type III < I
Fine collagen fibers organized into nodules, predominantly parallel	Large, thick collagen fibers, closely packed random to epidermis
Flatter collagen fibers in wavy pattern	Fibers lie haphazardly
High collagen cross-link	Collagen cross-link twice higher than in HS
Myofibroblasts that express α-SMA	Absence of myofibroblasts
Fibroblasts: ↑cell number, ↑↑proliferation, ↓↓apoptosis, ↑↑collagen I	↑↑proliferation, ↑↑collagen I
↑↑TGF-β1, ↑TGF-β2, ↓↓TGF-β3	↑↑TGF-β1, ↑↑TGF-β2, ↓↓TGF-β3

*↑, increase; ↓, decrease; SMA, smooth muscle actin; TGF-β, transforming growth factor-beta; HSs, hypertrophic scars*.

## Prevention is the Key

The most significant segment of an approach to hypertrophic scarring is its prevention. Before an elective surgery doctors should be informed if their patients have had previous problems with scarring. When performing surgery, incisions should follow Langer’s lines that correspond to the natural orientation of collagen fibers in the dermis and are parallel to the direction of the underlying muscle fibers (62). Incisions made parallel to Langer’s lines are known to heal better and produce less scarring than those that cut across (63). Also, it is important that all the incisions are closed with minimal tension that they do not cross joint spaces and that midchest incisions are avoided whenever possible (49). For the non-surgical wounds following trauma, it is crucial to debride contaminated ones and remove foreign bodies to minimize the inflammatory response; also, to promote adequate wound management with regular dressing changes to provide wound coverage and moist healing (57, 64). All the predisposed individuals presenting with any inflammatory skin problems as acne or deeper skin infections should be treated correspondingly to minimize inflammation (65). If we cannot avoid surgery in patients at a high risk of HS, immediate silicone-based products such as gels or sheeting with or without corticosteroid injections should be administered. Silicone sheeting is considered as the internationally recommended first-line option of scar management, which should be used after the wound has fully epithelialized (66). They should be applied for 12–24 h/day with daily washing for at least 2 months up to 1–2 years (54). Silicone gel is often preferred to sheeting from the perspective of ease of application and patient compliance especially when applied on the face, mobile areas, and in patients living in humid climates due to sheeting dislodgment. Silicone gel has been shown not to be inferior to sheeting in improving objective and subjective characteristics of scars, but it is superior in the ease of use (67). However, for extensive hypertrophic burn scars pressure garments still represent the first-line prophylactic therapy (68). The type of preventive scar measure that would be applied to a patient depends on the individual’s risk factors and his/her esthetic concerns. After trauma, in patients with moderate risk of scarring it is advised to apply silicone gels or dressings as preferred therapy, with topical products containing onion extracts or hypoallergenic adhesive tape for a few weeks after surgery as acceptable alternatives (69). In low-risk patients, we should just monitor wounds and prescribe silicone sheeting products for worried individuals (70). Other general measures to prevent HS formation include compression therapy, the use of moisturizers, manual massage, and strict UV photoprotection measures during scar formation and maturation phase to avoid hyperpigmentation (71, 72). As a rule, scars should be reevaluated 4–8 weeks after surgery to determine further management.

## Scar Evaluation

If eventually scar forms, regardless of whether or not prophylactic measures were applied, it should be evaluated. When assessing scars, their size, color, contour, height (thickness), surface area, surface texture, pliability, location, and subjective symptoms such as itching and pain, and also patient’s perception should be taken into account. It has been indicated that this subjective component of the patient’s view of the scar is as important as objective aspect and it may be very influential in determining the patient’s quality of life (73, 74). Assessment of the scars is a frequent topic of discussion among clinicians because there is no generally accepted evaluation tool, although various ones have been proposed (75–77). None of these, however, seem suitable as a stand-alone tool, suggesting that combination of objective imaging tools and scar scales and questionnaires may be justified to achieve comprehensive documentation in everyday clinical practice (78).

## Current Approach to HS Management

When dealing with future scar reduction modalities, it is of great importance for clinicians to not only discuss with patients their concerns, needs, and expectations but also to educate them about possible treatment options and their outcomes. The approach to treatment and its goals should be set for the individual patient based upon scar evaluation, patients’s characteristics, and expectations in order to reduce the scar volume, minimize subjective symptoms, i.e., pain and pruritus, and to improve function and esthetic appearance. As we have mentioned earlier, HSs are characterized by their ability to potentially regress over time. However, this maturation process is slow. So, the goal of the treatment is to stimulate this process to improve objective and subjective symptoms. According to updated international clinical recommendations on scar management (54) and others that adopted them (70), when treating linear or small HS resulting from trauma or surgery, silicone gel sheeting and topical onion extracts are considered as the first-line therapy. If there is no improvement within a month, we can start second-line therapy that is intralesional corticosteroid injections—triamcinolone acetonide 10–40 mg/mL with or without cryosurgery at monthly intervals for 3–4 months. Second-line therapy also includes laser therapy (pulsed-dye laser or fractional laser) and surgical excision in combination with postoperative silicone sheeting or postoperative intralesional corticosteroid/5-fluorouracil (5-FU) injections (79). Corticosteroids suppress healing and abnormal scarring by three mechanisms: vasoconstriction, anti-inflammatory and immunosuppressive effect, and inhibition of keratinocyte and fibroblast proliferation (80). When facing extensive HSs as are the postburn ones, of great importance is acute treatment at specialized burn centers where debridement and possible skin grafting are performed. Afterward, first-line therapy, as in linear HSs, includes silicone sheeting or gel and topical onion extracts. First-line also includes pressure garments that are also the first-line prophylactic therapy measure for postburn scars. Current international guidelines recommend ablative fractional laser, CO_2_ laser, as promising second-line therapy for this extensive HSs but also for inactive, linear HSs. The abovementioned commonly used and innovative scar-reducing modalities are presented in detail in Table 2.

**Table 2:** Treatment options for hypertrophic scars.

Therapeutic modality (application)	Mechanism of action	Advantages	Disadvantages	Comment	Reference
**Topical agents**					
Silicone gel Silicone sheet	Optimal occlusion and hydration of the stratum corneum; ↓TEWL, subsequent ↓cytokine-mediated signaling from keratinocytes to dermal fibroblasts. Gentle reduction of tension. Static electricity	Easy to use, can be applied at home Non-invasive, safe, tolerated by children Multiple formulations and formats available	Sheets need to be washed daily. Risk of infection 6–12 months constant wear to achieve optimum results. Expensive	Should be avoided on open wounds Gel preferred over sheets on visible areas and in hot climates For prevention of HS; treatment can be considered as additional therapy in active HS Poor study design	(66, 81–83)
Onion extract creams	Anti-inflammatory effect, bactericidal, and inhibit fibroblast proliferation Flavonoids (quercetin and kaempferol) in onion extract play the main role in reducing scar formation through inhibition of fibroblast proliferation Induction of MMP-1 Inhibition of TGF-β1 and -β2 and SMAD proteins Improve color, stiffness, and irregularity of the scar	Well-tolerated preventative treatment	Need for early initiation	Onion extract therapy should be used in combination with an occlusive silicon dressing to achieve a satisfying decrease in scar thickness. Now available in form of an occlusive patch that has dual effect	(84–87)
Imiquimod 5% cream (alternate night applications for 2 months after surgery)	↓TNF-α, INF-α, IL-1, IL-4, IL-5, IL-6, IL-8, IL-12, alters the expression of markers for apoptosis; improved scar quality	Minimal recurrence	May cause hyperpigmentation, irritation	Resting period from the treatments usually needed	(88, 89)

**Intralesional injections**					
Corticosteroid injections; TAC (10–40 mg/mL into papillary dermis every 2–4 weeks until scar is flattened)	Vasoconstrictive, anti-inflammatory, immunosuppressive effect. Inhibition of keratinocyte and fibroblast proliferation, glycosaminoglycan synthesis. ↓MMPs inhibitors	Inhibit the formation of HS. Reduce pain and pruritus	Multiple injections administered by a clinician. Discomfort, painful. Skin atrophy, telangiectasia, hypopigmentation	Monotherapy or in combination with two 15-s cryotherapy cycles prior to application to facilitate the injection through the development of edema, to reduce the pain and improve the result. Clinical benefit of adding 5-FU. TAC treatment can be performed on the day of surgery to prevent the formation of HS in patients at risk	(90–92)
5-FU 50 mg/mL Weekly intervals, 2- or 4-week intervals; 3–6 injections TAC:5-FU 4:45 mg/mL (1:9); 10:37.5 (1:3)	Cell proliferation inhibition, ↑ fibroblast apoptosis, collagen-1 suppression, MMP-2 induction	No systemic side effects	Pain, purpura, burning sensation, transient hyperpigmentation Risk of ulcerations in dark-skinned patients	Alone or with corticosteroids (more effective and less painful); combination of TAC (40 mg/mL) and 5-FU (50 mg/mL) (1:3) injected intralesionally once weekly for 2 months—superior to exclusive weekly injection of TAC 40 mg/mL The addition of the pulsed-dye laser treatments is to be most effective Not recommended during pregnancy, bone marrow suppression, anemia, etc. At the start of treatment as well as after four injections a blood count should be done	(93–95)
Interferon therapy (INF-α, β, γ) INF-α2b—3 times weekly INF-γ—intralesionally once per week up to a dosage of 0.05 mg for 10 weeks or 0.01–0.1 mg 3 times a week/3 week	↑Collagen breakdown, ↓TGF-β (Smad7 pathway), ↓ECM production, ↓ collagen I and III synthesis	No serious toxic effects Dermal cream containing liposome-encapsulated IFN-α2b	Painful when administered intralesionally. Flu-like symptoms. Expensive	Concept of the early topical use of this antifibrogenic agent for the treatment of dermal fibroproliferative disorders	(96, 97)
Bleomycin [intralesional multiple injections 0.1 mL (1.5 IU/mL) at a max dose of 6 mL, 2–6 sessions within a month]	Induces apoptosis, ↓TGF-β1—↓ collagen synthesis ↓Height, pliability as well as reduction in erythema, pruritus, and pain	Easy to administer, cheap, high regression rate, minimum complication and recurrence	Sporadically, development of depigmentation and dermal atrophy has been noted. Systemic toxic effects of intralesional injections appear to be rare	Considerable success. Due to its toxicity, clinicians are encouraged to be aware of associated potential problems Larger scale prospective studies needed	(98–100)
Verapamil (intralesional 2.5 mg/mL)	Stimulates procollagenase synthesis—↓collagen synthesis, ↑collagen breakdown, ↓scar elevation, vascularity, pliability	Low cost, fewer adverse effects		Monotherapy or as adjuvant therapy after excision with or without silicone	(101, 102)
Botulinum toxin A Intralesional injections (2.5 U/mL at 1-month intervals) for 3 months 4–7 days before the surgery	↓Erythema, itching sensation, and pliability Chemoimmobilization—temporary muscular paralysis, ↓tension vectors on wound edges, enhances scarring of facial wounds. ↓CTGF, ↓TGF-α1	Acceptable for both doctors and patients Improvement and the rate of therapeutic satisfaction is very high	Expensive	Beneficial for use in young patients for wounds without tissue loss, lying perpendicular to the reduced tension lines of the skin of the face Larger, randomized, control studies are warranted	(103–105)
TGF-β and isomers avotermin (hrTGFβ-3) (50–500 ng/100 μg per linear centimeter of wound margin given once)	Significant improvement in scar appearance	Safe and tolerable		Prevention or reduction of scarring following surgery. Ongoing clinical trials	(106–108)
Mannose-6-phosphate	Reduction of fibrosis by inhibiting TGF-β1 and 2 activation	Safe and tolerable		Clinical trial	

**Other current therapeutic options**					
Compression therapy					
Elastic bandages or pressure garments (20–40 mmHg)	Reduction in scar thickness MMP-9 activation; prostanglandin E2↑, subsequent ↑collagenases. Pressure-induced hypoxic effects leading to collagen and fibroblast degeneration	Non-invasive. Can be applied at home Recommended for special locations (e.g., on the ear)	Expensive (custom made). Poor compliance (cause discomfort; 6–24 months constant wear to achieve optimum results). Sweating and swelling of the limbs; dermatitis, pressure erosions, and ulcerations can develop	Treatment of postburn scars and scars in children. Applied when wound is closed. Can be used in combination with silicones. The beneficial effects remain unproven	(109–113)
Cryotherapy (monthly sessions)	Induce vascular damage that may lead to anoxia and ultimately tissue necrosis ↓Scar volume, hardness, elevation, erythema	Easy to perform, low cost	Hypopigmentation, pain, moderate atrophy, protracted healing time	Useful on small lesions. Easy to perform. New intralesional cryoneedles have shown ↑ efficacy	(95, 114)
Surgery Z- or W-plasty, grafts, or local skin flaps	Interrupt the circle between scar tension and ensuing further thickening of the scar due to permanently stimulated ECM production		Invasive. Risk of recurrence	Z-plasty option for burns. Immediate postsurgical additional treatment needed to prevent regrowth First-line treatment if disabling scar contractures are present. Surgical therapy of HS without tension and without contractures, present less than 1 year, is not recommended	(115)

**Laser procedures**					
Ablative lasers (CO_2_, Er:YAG)	Induction of capillary destruction—generates hypoxemia—alters local collagen production. ↑MMPs Improvement of pigmentation, vascularity, pliability, and scar height	Reach greater depths than a pulsed-dye laser	Mild side effects that include a prickling sensation during treatment and post-treatment erythema Erosions, weeping, and crusting can occur	For inactive HS with height differences, bridge or contracture formation. CO_2_ shows superior effectiveness. Fractional CO_2_ is option in postburn HS	(116)
Non-ablative lasers; pulsed-dye laser 585/595 nm	Induction of selective capillary destruction—generates hypoxemia—alters local collagen production. ↑MMPs	Minimal side effects, purpura usually persisting for 7–14 days	Expensive. Specialist referral needed. Vascular-specific	Excellent first-line treatment, preventive strategy for HS, reduce erythema primarily	(98, 117, 118)
Gold standard: application on the day of suture removal, 44.5 J/cm^2^ about 1.5–2 ms (every 3–4 weeks)	Reducing erythema, pruritus, pliability, improving skin texture	Depending on the energy density employed, vesicles and crusts may occur		Do not appear to be adequate for thick HS	

*↑, increase; ↓, decrease; TEWL, transepidermal water loss; ECM, extracellular matrix; MMP, matrix metalloproteinase; HS, hypertrophic scar; CTGF, connective tissue growth factor; IL, interleukin; 5-FU, 5-fluorouracil; TAC, triamcinolone acetonide; TGF-β, transforming growth factor-beta*.

## Conclusion

Scarring and its accompanying esthetic, functional, and psychological sequelae still pose major challenges. To date, there is no satisfactory prevention or treatment option for HS, which is mostly due to not completely comprehending the mechanisms underlying their formation. A predominant role in hypertrophic scarring prevention and treatment still maintain silicone sheeting or gel. The efficacy and safety of this gold-standard, non-invasive therapy has been demonstrated in many clinical studies, but to date, exact mechanisms by which they improve HS are yet to be fully agreed upon. Second most validated and more specialized scar treatment is intralesional corticosteroid injections, especially in combination with other therapeutic modalities like 5-FU, which augment the result and reduce the side effects of corticosteroids. Current therapeutic approaches with their empirical effects are unreliable and unpredictable. Therefore, there is an unmet need for an effective, targeted therapy and prevention, which would be based on an action or a modulation of a specific factor with clarified mechanism of action that has a beneficial effect on wound healing. As the ECM is involved in cellular and extracellular events that lead to pathological scarring, targeting its components mostly by regulating BMPs may throw up new therapeutic approach for reduction or prevention of pathological scarring or HSs with functionally and cosmetically acceptable outcome.

## Author Contributions

ZM and AJ performed the literature review and wrote the manuscript. ID-Č participated in literature search and review. KK and RČ provided assistance in preparation of the tables. LG and BM revised the manuscript critically. All authors read and approved the final version of the submitted manuscript.

## Conflict of Interest Statement

The authors declare that the research was conducted in the absence of any commercial and financial relationships that could be construed as a potential conflict of interest.
